# Effect of 2‐methoxyestradiol on mammary tumor initiation and progression

**DOI:** 10.1002/cnr2.2068

**Published:** 2024-04-10

**Authors:** Kimberly T. Peta, Chrisna Durandt, Marlene B. van Heerden, Anna M. Joubert, Michael S. Pepper, Melvin A. Ambele

**Affiliations:** ^1^ Department of Immunology, Institute for Cellular and Molecular Medicine, South African Medical Research Council, Extramural Unit for Stem Cell Research and Therapy, Faculty of Health Sciences University of Pretoria Pretoria South Africa; ^2^ Department of Oral and Maxillofacial Pathology, School of Dentistry, Faculty of Health Sciences University of Pretoria Pretoria South Africa; ^3^ Department of Physiology, School of Medicine, Faculty of Health Sciences University of Pretoria Pretoria South Africa

**Keywords:** 2‐methoxyestradiol, breast cancer, in vivo, metastasis, tumor growth

## Abstract

**Background:**

The anti‐cancer agent 2‐methoxyestradiol (2‐ME) has been shown to have anti‐proliferative and anti‐angiogenic properties. Previously, the effect of 2‐ME on early‐ and late‐stage breast cancer (BC) was investigated in vivo using a transgenic mouse model (FVB/N‐Tg(MMTV‐PyVT)) of spontaneous mammary carcinoma. Anti‐tumor effects were observed in late‐stage BC with no effect on early‐stage BC. Given the contrasting results obtained from the different BC stages, we have now investigated the effect of 2‐ME when administered before the appearance of palpable tumors.

**Methods:**

Each mouse received 100 mg/kg 2‐ME on day 30 after birth, twice per week for 28 days, while control mice received vehicle only. Animals were terminated on day 59. Lung and mammary tissue were obtained for immunohistochemical analysis of CD163 and CD3 expression, and histological examination was performed to analyze tumor necrosis. Additionally, blood samples were collected to measure plasma cytokine levels.

**Results:**

2‐ME increased tumor mass when compared to the untreated animals (*p* = .0139). The pro‐tumorigenic activity of 2‐ME was accompanied by lower CD3+ T‐cell numbers in the tumor microenvironment (TME) and high levels of the pro‐inflammatory cytokine interleukin (IL)‐1β. Conversely, 2‐ME‐treatment resulted in fewer CD163+ cells detectable in the TME, increased levels of tumor necrosis, increased IL‐10 plasma levels, and low IL‐6 and IL‐27 plasma levels.

**Conclusion:**

Taken together, these findings suggest that 2‐ME promotes early‐stage BC development.

## 1 INTRODUCTION

Breast cancer (BC) is one of the most frequently occurring cancers.^1^, ^2^ It is the most prevalent cancer among women worldwide, accounting for nearly 25% of all cancer cases in women according to the World Health Organization (WHO).^1^ Surgery is usually the first treatment option for and can include either lumpectomy or mastectomy.^3^, ^4^, ^5^ Radiation therapy is often used to eliminate cancer cells that have escaped surgery.^3^, ^4^, ^5^ Hormonal therapy is used to prevent estrogen from promoting the growth of certain types of BC.^3^, ^4^, ^5^ Chemotherapy is a systemic treatment that primarily inhibits DNA synthesis and mitosis leading to apoptosis in rapidly driving cancer cells.^6^ Depending on the stage of the cancer, chemotherapy may be administered before or after surgery.^3^, ^4^, ^5^

Advances in the development of chemotherapy include more targeted treatments that specifically target proteins involved in cancer cell growth and progression in cancers including BC, ovarian cancer, and prostate cancer.^6^ 2‐Methoxyestradiol (2‐ME) targets the colchicine‐binding site in tubulin and alters polymerization kinetics hindering tumor vascularization and growth.^7^, ^8^ Similar to 2‐ME, benzopyran derivatives, such as K‐1 compound exert their anti‐estrogenic effects by competitively inhibiting oestradiol binding to estrogen receptors.^9^, ^10^, ^11^ This shared mechanism of action underscores their potential as therapeutic agents in BC by mitigation of estrogen‐driven tumor growth and progression.^12^, ^13^ Numerous studies in BC, both in vitro and in vivo. have demonstrated that 2‐ME has anti‐angiogenic and anti‐proliferative properties.^14^, ^15^, ^16^, ^17^, ^18^, ^19^, ^20^, ^21^ Moreover, the use of 2‐ME has been shown to enhance the effects of chemotherapy in the treatment of BC.^22^ For example, combining 2‐ME with paclitaxel, a widely used chemotherapy drug, has been shown to improve treatment outcomes in preclinical models of BC.^22^ Despite its promising anti‐tumor properties, more research is required to fully comprehend the potential benefits and risks of 2‐ME in BC treatment.

Cytokines are generated by different cell types present in the TME and contribute to a complex, dynamic system by facilitating crosstalk between the different cell types. The majority of the cytokines are produced by macrophages and infiltrating T‐helper (Th) cells.^23^, ^24^ The Th subsets, Th1 and Th2 are antagonistic to each other and produce cytokines that initiate different activities.^23^, ^24^ Th1 cells effectively generate an anti‐tumor immune response by secreting granulocyte macrophage colony‐stimulating factor (GM‐CSF), interferon gamma (IFN‐γ), IL‐2, IL‐12 and tumor necrosis factor alpha (TNF‐α) that stimulate cytotoxic lymphocytes and macrophages (M1) thereby promoting inflammation and cellular immunity.^23^, ^24^ Th2 cells produce IL‐4, IL‐5, IL‐13, chemokine ligand 2 (CCL2) also referred to as monocyte chemoattractant protein 1 (MCP‐1), CCL7, and CCL11 that stimulate antibody production by B cells and M2 macrophage polarization that are essential in mediating a humoral immune response reported to enhance mammary tumor development.^23^, ^24^ A recent study has revealed that B and T lymphocytes can indirectly exert pro‐tumor activity by regulating the bioactivity of myeloid cells such as monocytes, macrophages and mast cells leading to metastasis and resistance to endocrine therapies.^25^ Chemotherapy drugs act non‐specifically on various cell types in the TME, influencing cytokine production by these cells, hence affecting tumor growth and progression. For example, paclitaxel, a chemotherapeutic drug, is a microtubule destabiliser that is used to treat BC.^26^ Paclitaxel increases IL‐1β, IL‐10, IL‐6, and IL‐8 in the plasma.^26^, ^27^ However, this drug decreased the level of TNF‐α.^28^ Furthermore, paclitaxel increases T cell (CD3+) activation in BC patients and increased T‐cell clones inside tumors.^29^, ^30^ This indicates that multiple cytokines and leukocytes are present in the BC tumor microenvironment (TME).^23^, ^25^

The number of CD3+ T‐cells and M2‐associated CD163 macrophages in the TME are prognostic factors in BC patients.^31^ Following antigen recognition, CD3 (T‐cell receptor) initiates a signaling cascade that activates both CD4+ and CD8+ T cells,^32^ which are referred to as tumor infiltrating lymphocytes (TILs) and are involved in eliminating tumor cells.^33^, ^34^ Increased numbers of CD3+ T cells in the TME are associated with small tumor size, decreased lymph node metastasis and increased overall BC patient survival.^33^, ^35^, ^36^ Conversely, increased numbers of CD163+ macrophages are associated with poor prognosis, large tumor size, metastasis, distant recurrence, tumor progression, and decreased survival.^37^, ^38^, ^39^, ^40^

Numerous studies have revealed the antitumour impact of 2‐ME on BC progression, predominantly utilizing xenograft models. In light of this, we previously examined the effect of 2‐ME on early‐ and late‐stage mammary carcinoma in a transgenic mouse model (FVB/N‐Tg(MMTV‐PyVT)) that spontaneously develops mammary carcinogenesis.^41^ 2‐ME treatment was initiated (i) as soon as palpable tumors appeared for early‐stage BC investigation or (ii) on day 28 after the appearance of palpable tumors for late‐stage BC investigations. 2‐ME treatment of late‐stage BC inhibited mammary tumor growth and slowed pulmonary metastasis, while a pro‐tumor effect was observed in early‐stage BC.^41^ This contrasting effect of 2‐ME on early‐ and late‐stage BC may suggest that the anti‐tumor effect of 2‐ME may be dependent on the stage of mammary carcinoma, thus prompting the initiation of this study. This study therefore aimed to investigate the effect of 2‐ME on tumor initiation in FVB/N‐Tg(MMTV‐PyVT) transgenic mouse model. This was done to investigate the BC stage‐specific effect of 2‐ME treatment in order to understand the effect of 2‐ME on BC development and progression, and to further determine at exactly what BC stage 2‐ME would be beneficial as a treatment option. Furthermore, this study will provide data on the potential of 2‐ME as an effective preventive treatment in a scenario where BC development is evident such as in hereditary BC.

## 2 MATERIALS AND METHODS

### 2.1 Animal studies

This study was approved by the University of Pretoria Faculty of Health Sciences Research Ethics Committee (ethics reference no.: REC166‐19) and the Animal Ethics Committee (ethics reference no.: 534/2019). The FVB‐TgN(MMTV‐PyVT) mouse model was obtained from Jackson Laboratory (Bar Harbor, ME, USA), and mice were bred to produce heterozygous offspring by crossing hemizygous males with wild‐type females. All offspring were genotyped, and only heterozygous females were used in the study.

### 2.2 Animal genotyping

The KAPA Mouse Genotyping Kit (KAPABIOSYSTEM, Cape Town, South Africa) was used to genotype mice according to the manufacturer's instructions. A 2 mm mouse tail biopsy was placed in 0.2 mL microcentrifuge tubes, and DNA was extracted. Two primer pair sequences obtained from the Jackson Laboratory website (The Jackson Laboratory; Bar Harbor, ME, USA) were used for polymerase chain reaction (PCR) genotyping experiments. The forward primer 5′‐GGAAGCAAGTACTTCACAAGGG‐3′ and reverse primer 5′‐GGAAAGTCACTAGGAGG‐3′ were specific for the transgene, while the forward primer 5′‐CAAATGTTGTCTGGTG‐3′ and reverse primer 5′‐GTCAGTCGAGTGCACAGTTT‐3′ were specific for the internal positive control. A thermocycler (GeneAmp® PCR System 9700, CA, USA) was used to amplify DNA under the following conditions: initial denaturation at 95°C for 3 min, denaturation at 95°C for 3 min, denaturation at 95°C for 3 min, denaturation at 95°C for 3 min, denaturation at 95°C for 15 s, annealing at 60°C for 15 s, and extension at 2 min for 35 cycles. The amplicon sizes were determined using 2% agarose gel electrophoresis stained with ethidium bromide (10 mg/mL).

### 2.3 2‐Methoxyestradiol treatment and tumor measurements

In this experiment, mice (30 days of age) were given 100 mg/kg of 2‐ME in a vehicle consisting of 90% sunflower oil (Sunfoil, South Africa) and 10% dimethyl sulfoxide (DMSO) two times per week via oral gavage for 4 weeks before being euthanized. The vehicle alone was given to control mice. Treatment was administered a total of eight times and began on day 30 after birth, which is before palpable tumors appeared. On average, palpable tumors appeared at day 50 after birth as determined in our laboratory (data not shown). Mammary tumors were excised, and the mass measured on a scale (Sartorius, Göttingen, Germany) at termination in grams (g). A light microscope (OLYMPUS 100, Dubai, UAE) was used to identify and count the number of lesions on the lung surface. CellSens dimension imaging software (XV imaging, product version 3.9, Hague, Netherlands) was used to process images. Eighteen heterozygous female mice (nine for 2‐ME‐treated and nine for control) were used.

### 2.4 Histology and immunohistochemistry

Lung and mammary tissues were collected from both euthanized 2‐ME treated and control group mice and fixed in 10% neutral buffered formalin. Hematoxylin and eosin (H&E) staining was performed as previously described,^42^, ^43^ and immunohistochemical analysis for CD163 and CD3 staining was conducted with minor modifications as previously described.^42^, ^43^ Formalin‐fixed paraffin‐embedded (FFPE) tissue blocks were cut into 3‐micron sections and baked overnight at 58°C. After deparaffinizing the slides in xylene, they were hydrated with decreasing concentrations of alcohol to distilled water. Endogenous peroxidase was quenched for 5 min at 37°C with a 3% hydrogen peroxide solution. After antigen retrieval with a high pH buffer retrieval solution (Dako Envision FLEX Retrieval solution high pH, Agilent Technologies, Denmark), background staining was blocked for 30 min at room temperature with a protein block (Novolink Leica Biosystems, Newcastle Upon Tyne, UK). The sample sections were washed in phosphate buffer saline (PBS) after being incubated overnight at 4°C in a 1:300 anti‐CD163 antibody solution [EPR19518] (ab182422) (Abcam, Cambridge, UK). The antigen–antibody binding site was identified using the NovolinkTM Polymer Detection Kit (Leica Biosystems) as directed by the manufacturer. For chromogen detection, the slides were washed in PBS and incubated with 3,3′‐diaminobenzidine (DAB) (NovolinkTM Polymer Kit). Sections were washed and counterstained in hematoxylin for 1 min before being dehydrated in increasing concentrations of alcohol, cleared in xylene, and mounted with dibutylphthalate polystyrene xylene (DPX). CD3 IHC was performed on 3‐micron sections in the same manner as CD163, but with a few differences. Using a low pH buffer, the antigen was retrieved (Cell Conditioning Solution CC2, Ventana Medical Systems, Inc., Arizona USA). Sections were incubated in a 1:100 rabbit monoclonal anti CD3 (Abcam ab16669 clone SP7) antibody solution for 120 min at room temperature. Slides were rinsed in PBS and detected with anti‐rabbit Polymer HRP IgG for 30 min at RT (NovolinkTM Polymer Detection Kit, Leica Biosystems). PBS was used as a negative control instead of CD3 or CD163 antibody. The Leica AT 2 Aperio scanner (Leica Biosystems, Nussloch, Germany) was utilized to capture images at 40× magnification, and Qupath software (The Queens University of Belfast, Northern Ireland), version 0.3.2, was used for analysis. The software was used to import each scanned tissue section, and the image type was set to brightfield (H‐DAB). To focus solely on the cells within the section, a perimeter was drawn around the tissue section. The estimation stain vector was set to automatic to distinguish between positive and negative cells. The software was trained by identifying positively stained cells and indicating to it what constitutes positive cell detection. The detection image parameter was changed to “optical density sum,” and the scan was initiated. The final result displayed the number of positive, negative, and total cell counts. The percentage of CD163 and CD3 positive cells was calculated by dividing the number of positive cells by the total number of cells. The magnification of the images was 100 μm (immunohistochemistry), 500 and 800 μm (histology).

### 2.5 Measurement of plasma cytokines

Mice were humanely euthanized with isoflurane (Isofor; Piramal I Healthcare, Mumbai, India), and 800 μL of blood was collected via cardiac puncture in ethylenediaminetetraacetic acid (EDTA) tubes and centrifuged at 28766×*g* for 15 min. Aliquots of plasma (250–300 μL) were placed in 2 mL microcentrifuge tubes. The Legendplex mouse inflammation panel (13‐plex) kit (Biolegend®, San Diego, CA, USA) was used for cytokine profiling. The kit tests for 13 mouse cytokines: MCP‐1, GM‐CSF, TNF‐α, IFN‐γ, IFN‐β, IL‐1α, IL‐1β, IL‐6, IL‐10, IL‐12p70, IL‐17A, IL‐23, and IL‐27. The assay was carried out in accordance with the manufacturer's instructions. In summary, increasing standard concentrations (supplied with kit) were prepared (in duplicate) in a 96‐well plate to generate a standard curve. Analyses were done using a Cytoflex flow cytometer (Beckman Coulter, California, USA), ‘and Biolegend's data analysis software (https://legendplex.qognit.com/) was used to calculate cytokine concentrations.

### 2.6 Statistical analysis

For statistical analysis, GraphPad Prism version 5 (GraphPad Software Inc., San Diego, CA, USA) was used. A parametric one‐tailed unpaired *t* test was used to compare means between two groups. Data is presented as the mean plus standard error of the mean (SEM). A multiple comparison test and a two‐way ANOVA were used to compare the means of more than two categories.

## 3 RESULTS

### 3.1 Effect of 2‐ME on the rate of tumor appearance

**FIGURE 1:**
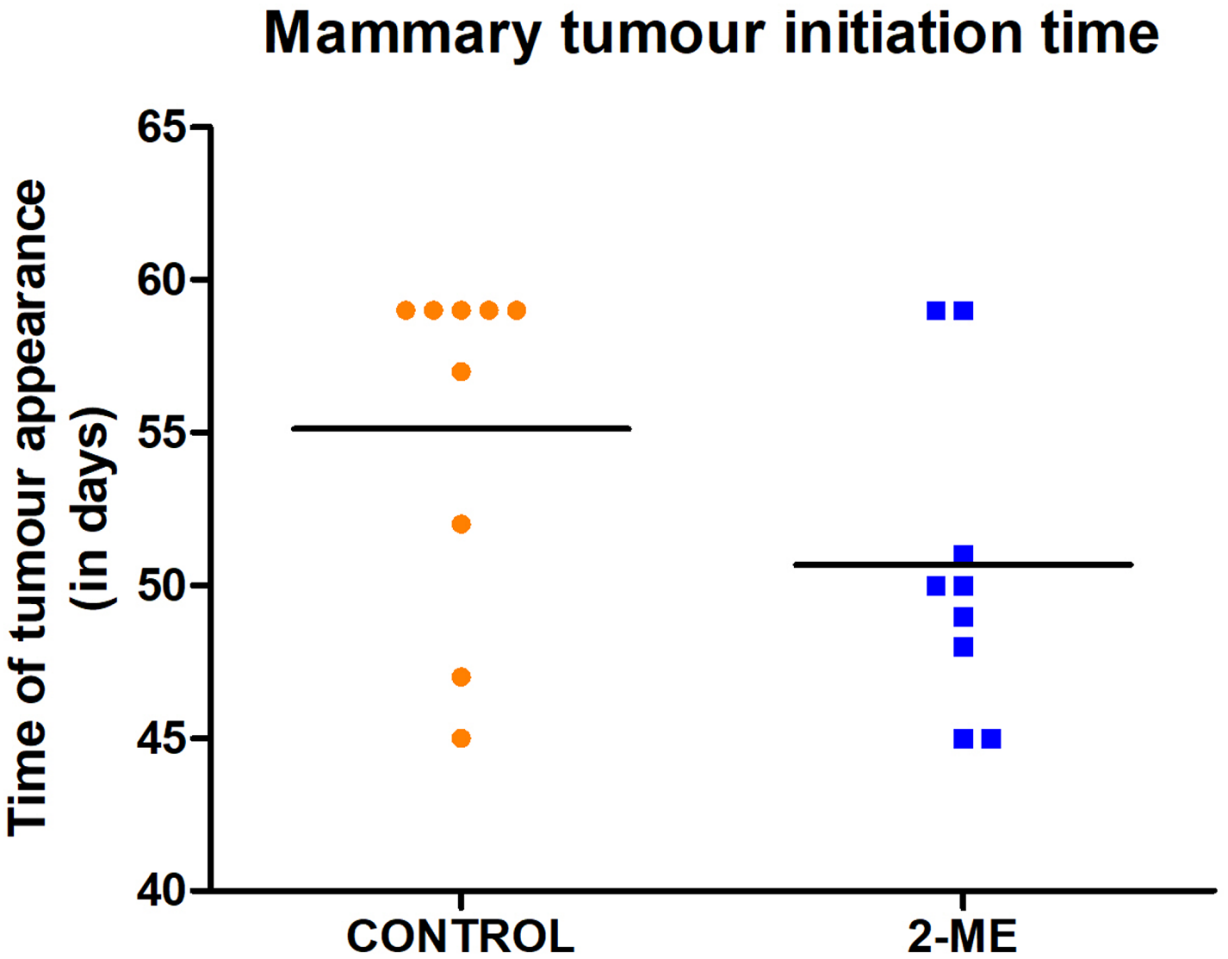
The time taken for mice to develop palpable mammary tumors. In the 2‐methoxyestradiol (2‐ME) and control groups, seven mice as opposed to three mice in the control group developed tumors earlier, respectively.

Seven mice in the 2‐ME group and three mice in the control group developed palpable tumors earlier (between day 45 and day 52), while two mice and six mice in the 2‐ME and control groups respectively, developed tumors later (between day 53 and day 59) (Figure 1). We observed that mice in the 2‐ME group exhibited the onset of palpable mammary tumors at an earlier stage compared to the control group, although the observed difference was not statistically significant.

### 3.2 Effect of 2‐ME on tumor volume and mass

**FIGURE 2:**
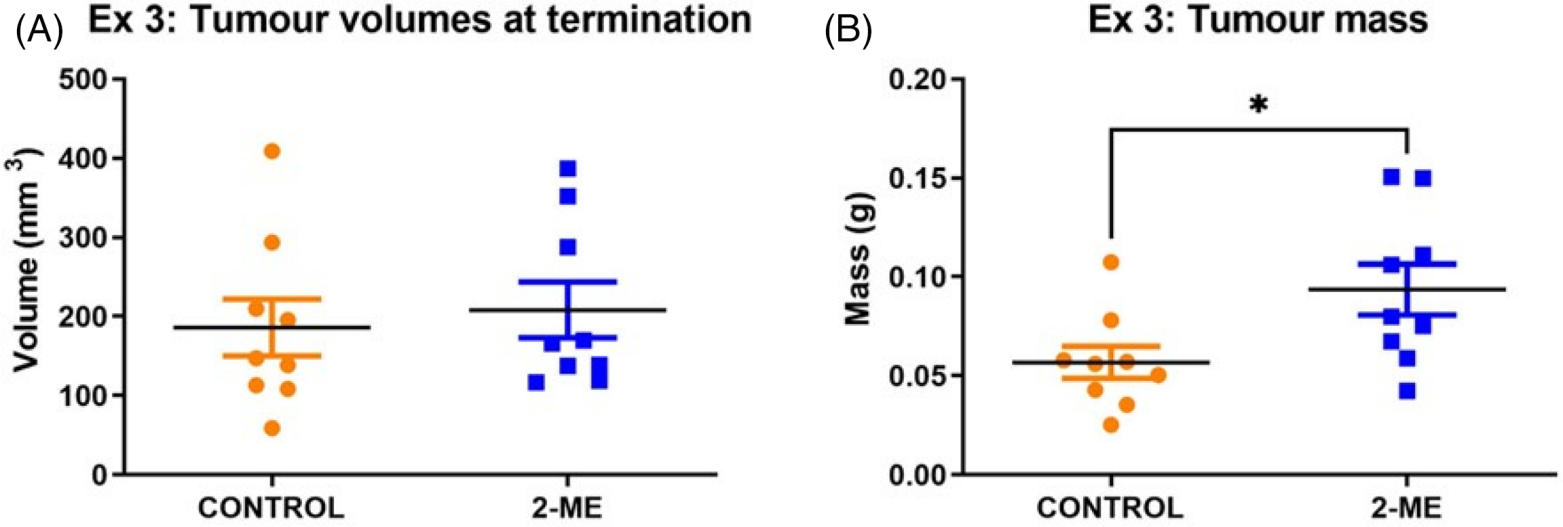
(A) No difference was observed in tumor volumes in the 2‐methoxyestradiol (2‐ME) treated and control groups (*p* = .3319). (B) A significantly greater tumor mass was observed in 2‐ME treated mice (*p* = .0139; *p* < .05) (*N* = 9 in each group).

The volume and mass of mammary tumors were measured at the time of termination. On average, no difference was observed in tumor volumes (Figure 2A), but tumor mass was significantly higher (*p* = .0139) in the 2‐ME treated group compared to the control group (Figure 2B). Importantly, the mammary tumor volumes and masses of each mouse were totaled; the plotted dots thus represent the total tumor volumes and masses of each mouse.

### 3.3 Histopathological analysis of mammary and lung tissues

**FIGURE 3:**
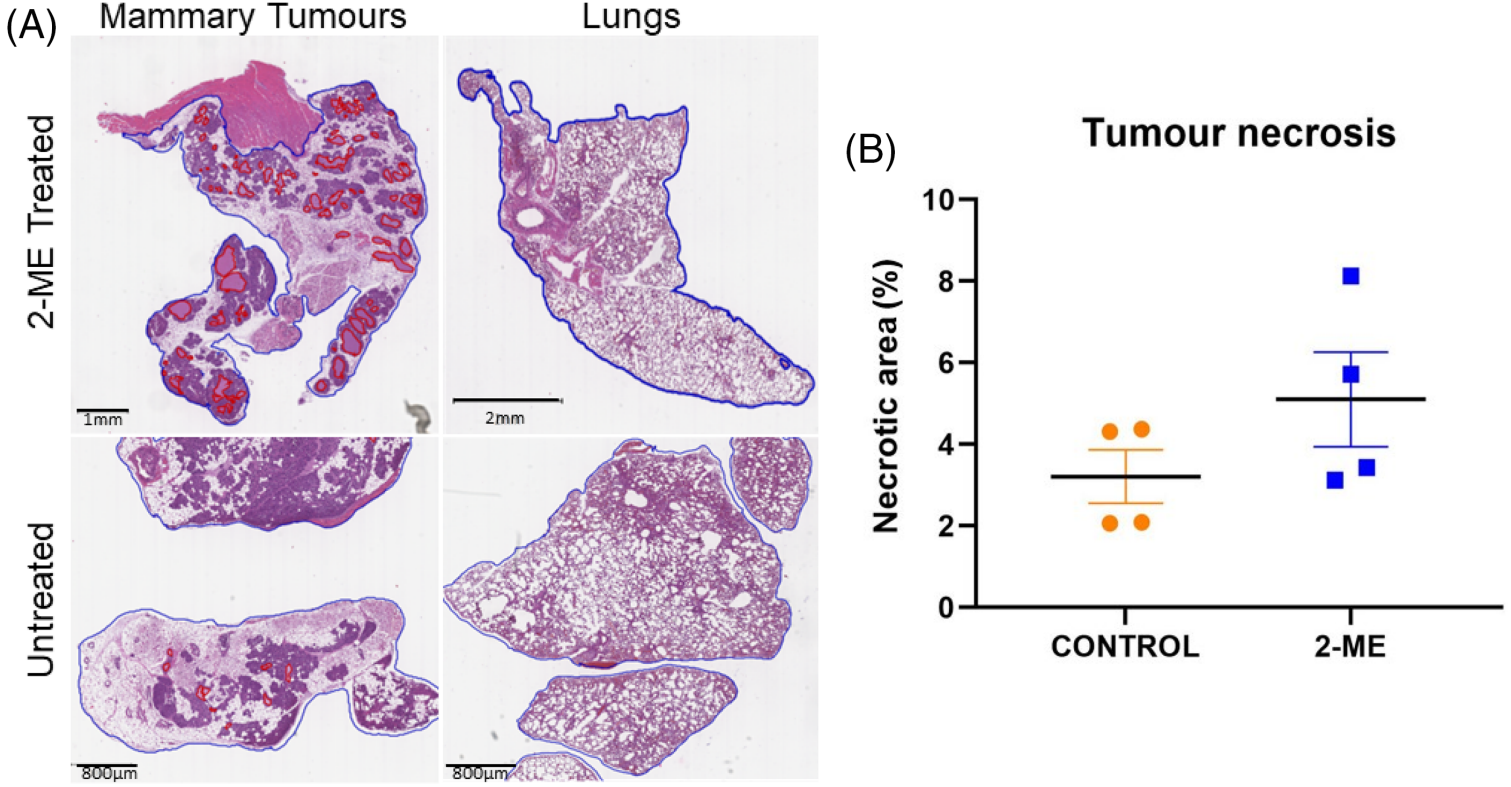
(A) Blue boundaries surround the tumor and necrotic regions are surrounded by red boundaries and arrows. Histology images showcasing various sizes (1, 2 mm and 800 μm) along with a scale bar. (B) More extensive (*p* = .1031) necrotic regions were observed in mice that were treated with 2‐methoxyestradiol (2‐ME) (*N* = 4).

Mammary tumor and lung tissues from 2‐ME treated and control groups were stained with H&E. Blue boundaries were drawn around the entire tissue region while red lines surround necrotic regions, and the arrow heads point to smaller necrotic regions (Figure 3A). Tumor necrotic tissue was greater in the 2‐ME treated group compared to the control group (Figure 3B). The result was, however, not statistically significant. Pulmonary necrosis was minimal and was detected in two mice from each group with similar necrotic area percentages. The lack of difference observed was likely due to premature termination, i.e. animals were terminated before pulmonary metastasis had time to occur.

### 3.4 Immunohistochemical analysis of M2 associated CD163 macrophages

**FIGURE 4:**
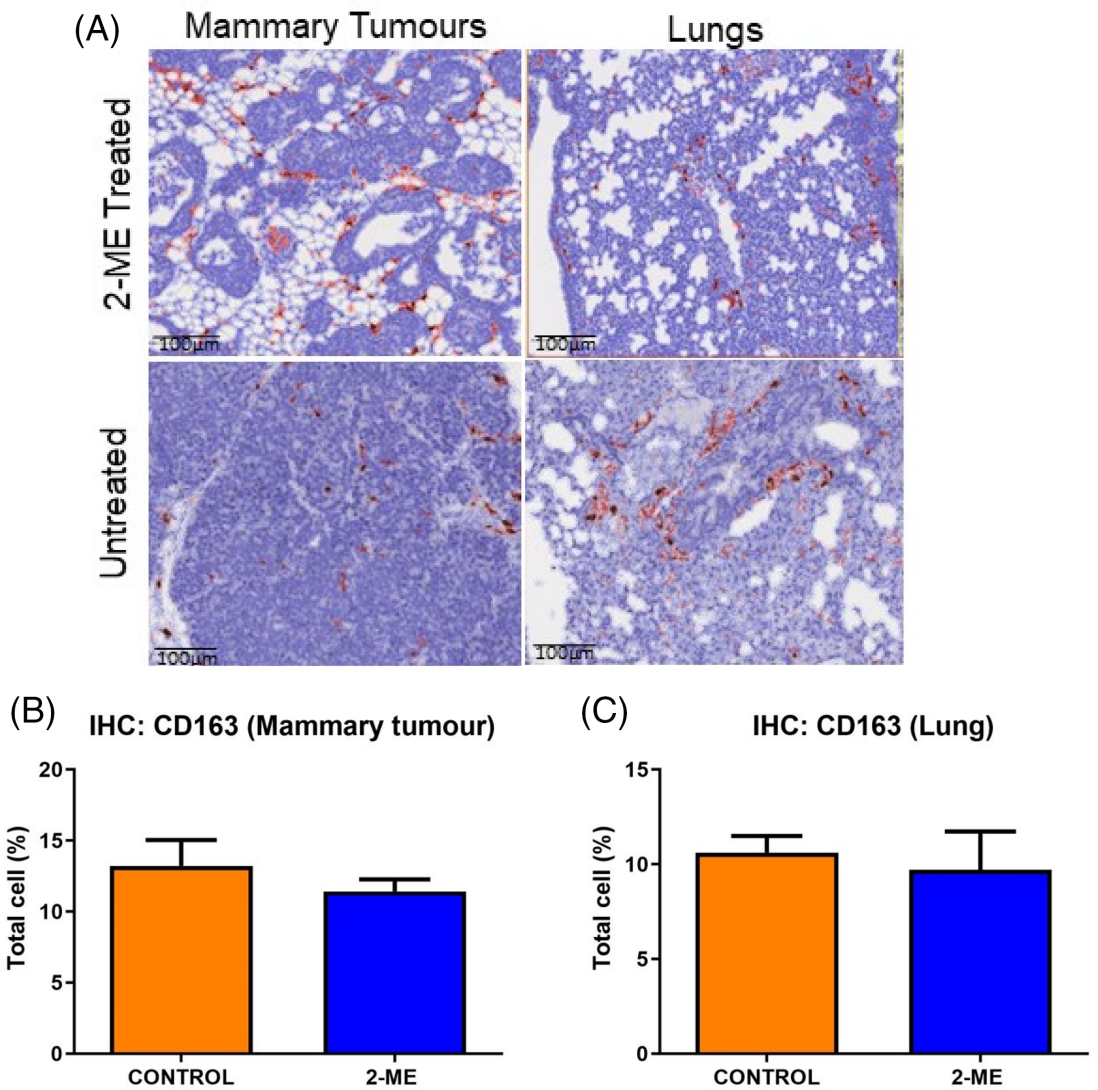
(A) CD163+ cells stained dark brown with red boundaries. IHC images accompanied by a 100 μm scale bar for reference. (B) A lower number of CD163+ cells (*p =* .1965) was detected in the 2‐ME group while (C) no change was observed in the lungs (*p* = .3450). *N* = 5 in each group.

Immunohistochemistry was performed on tumors from mammary and lung tissues in each group (2‐ME treated and control group). The CD163+ cells stained dark brown (Figure 4A). A lower number of CD163+ cells were detected in the mammary tumors of 2‐ME treated mice compared to controls, while no change was observed in the lungs (Figure 4B,C).

### 3.5 Immunohistochemical analysis of CD3 positiveCD3 positive cells

**FIGURE 5:**
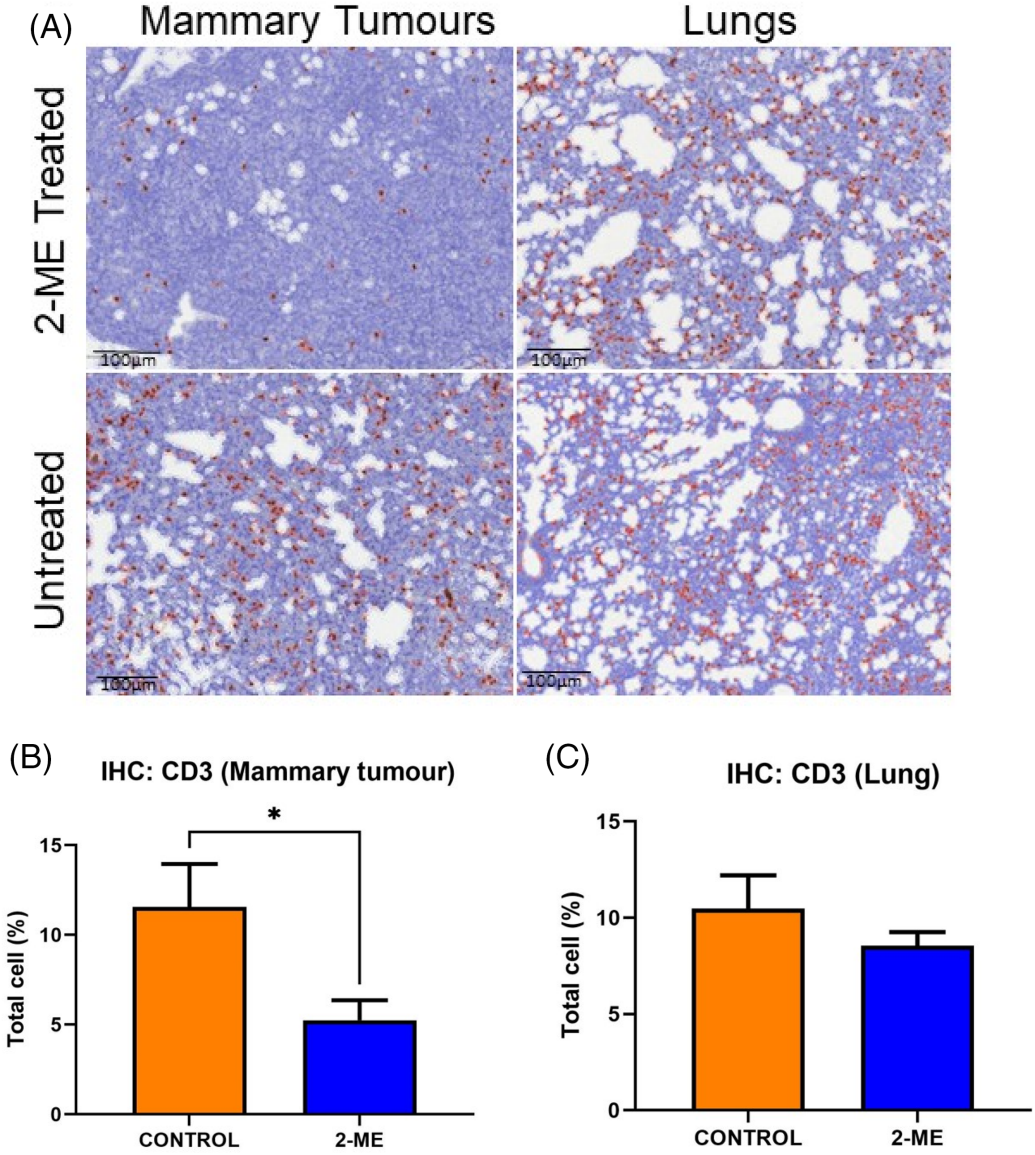
(A) CD3 immunohistochemistry in mammary and lung tissue. Immunohistochemistry (IHC) images with a scale bar representing 100 μm. (B) Significantly lower number of CD3+ T cells were observed in mammary tissue in the 2‐methoxyestradiol (2‐ME) group (*p* = .0217) (C) and lower number of CD3+ T cells were detected in the lung tissues of the same group. *n* = 5 in each group.

The dark brown stained cells indicate CD3+ T cells (Figure 5A). The number of CD3+ T cells was significantly lower in the mammary tumors of the 2‐ME group (*p* = .0217; Figure 5B). Similarly, fewer CD3+ T cells, although not significantly different compared to the control group, were observed in the lungs of the 2‐ME‐treated group (Figure 5C).

### 3.6 Cytokine profile

**FIGURE 6:**
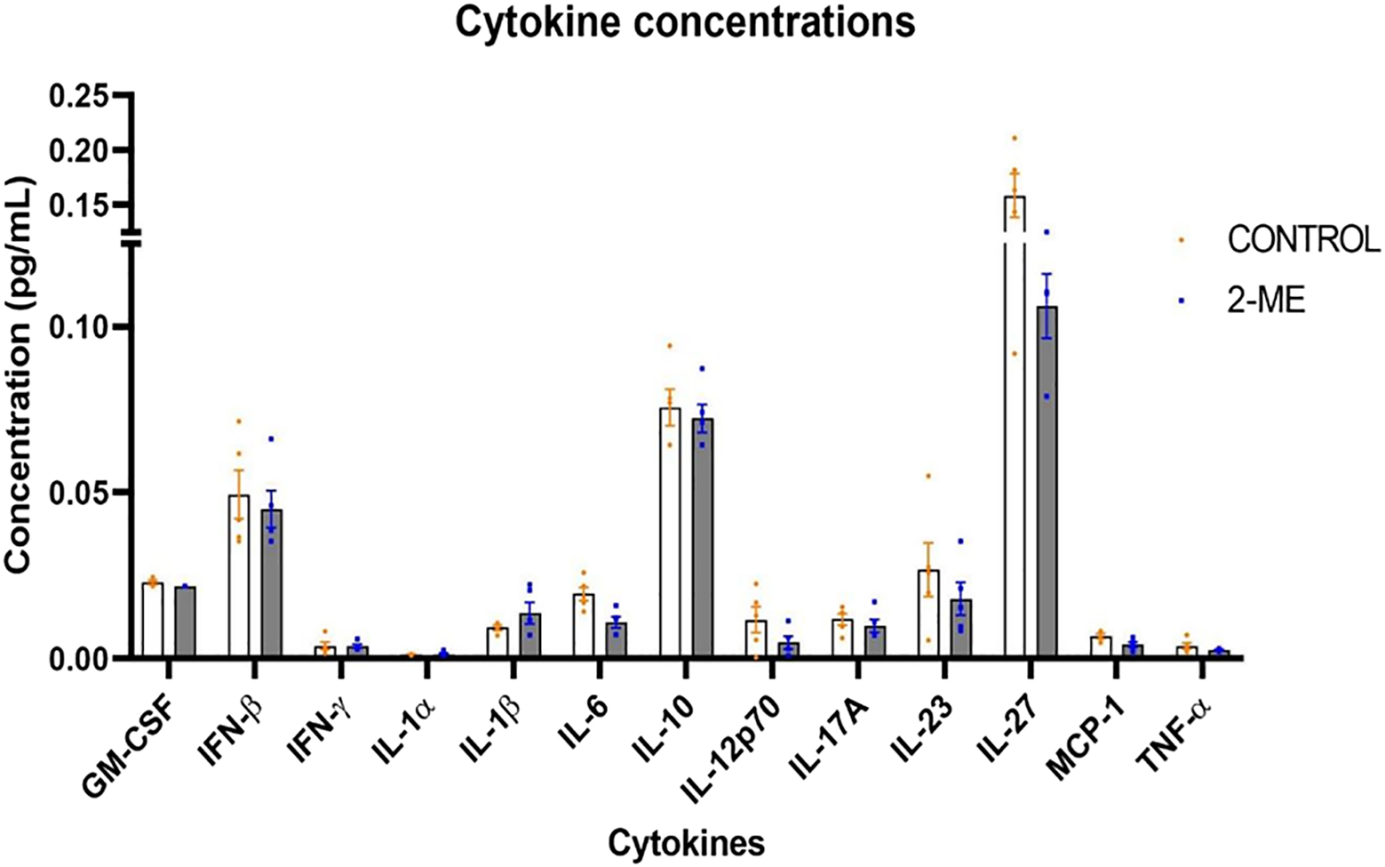
Cytokine profiles of 2‐methoxyestradiol (2‐ME) treated and control group (*n* = 5 in each group). IL‐6 was significantly higher (*p* = .0057; *p =* .05), and IL‐27 (*p* = .3317) were notably higher in the control group.

Plasma cytokine levels were measured in both 2‐ME treated and untreated mice. The cytokine concentrations that were similar in both groups were IL‐1α (*p* = .2063), IFN‐γ (*p* = .4562), TNF‐α (*p* = .1206), IL‐17A (*p* = 2408) and GM‐CSF (0.0526) Cytokine levels that were lower in the 2‐ME treated group include IL‐23 (*p* = .1914), IL‐12p70 (*p* = .0776), IL‐10 (*p* = .3183) and IFN‐β (*p* = .3231). The 2‐ME treated group had higher levels of IL‐1β (*p* = .2182). Notably, high levels of IL‐6, and IL‐27 were observed in the control group (Figure 6).

## 4 DISCUSSION

In this study, the effect of 2‐ME was investigated when administered before the development of mammary carcinoma in a MMTV‐PyVT transgenic mice that spontaneously develop palpable tumors. The mice received 2‐ME (100 mg/kg) treatment via oral gavage twice per week for a period of 4 weeks while the control animals received the vehicle alone. By day 51, more mice in the 2‐ME group (*n* = 7) compared to the control group (*n* = 3) developed early palpable mammary tumors. The findings suggest that 2‐ME may be involved in promoting early mammary tumor development. Huh et al. reported a similar finding whereby enhanced tumor multiplicity and growth were observed after 2‐ME treatment.^14^ The significantly lower number of CD3+ T cells observed in the 2‐ME group (*p* = .0217) may have contributed to the significant (*p* = .0139) tumor mass increase. High intertumoral CD3 is associated with good prognosis because of its cytotoxic activity.^33^, ^44^ Therefore, the low CD3+ T cell number indicates a protumor effect. 2‐ME has been reported to decrease CD3+ T cell proliferation but does not affect the cytokine production of T cells.^45^ Tumor necrosis was observed in the 2‐ME group which could be as a result of the lower CD163+ cell numbers present in the TME. A low number of CD163+ cells is associated with greater overall patient survival.^46^ It is also possible that the high levels of necrosis observed in the tumors of mice given 2‐ME may be more related to the drug's anti‐angiogenic effects previously reported,^14^ rather than the low number of CD163+ cells. 2‐ME may have reduced the blood supply to the tumor by inhibiting angiogenesis, resulting in hypoxia and subsequent necrosis.^47^ However, the expression of angiogenic markers was not investigated in this study and should be considered in the future. It is also plausible that together with 2‐ME activity, other immune cells such as neutrophils may be implicated in necrosis.^48^

Pulmonary metastasis was also investigated. The 2‐ME group had no lung lesions, while two mice in the control group had one lung lesion each. This is likely because mice were terminated early. Based on the Jackson laboratory reports, about 94% of female mice develop pulmonary metastasis by 3 months.^49^ Another study observed that pulmonary metastasis in this model occurred after 10 weeks.^50^ Only two mice, 1 from each group, had similar levels of necrotic tissue in the lungs. Both groups had a similar number of M2‐associated CD163+ cells. The most plausible explanation is that the termination timepoint on day 59 is shorter than when pulmonary metastasis is anticipated in this model. The number of CD3+ T cells were fewer, although not significantly, in the lungs of the 2‐ME group when compared to the control group. As previously mentioned, 2‐ME inhibits CD3+ T cell proliferation.^45^ Interestingly, Cimino‐Mathews et al found that fewer CD3+ T cells are associated with metastatic BC, a finding that is also aligned with our observation.^51^ In this study, animals were terminated at the beginning stages of metastasis (before 10 weeks) resulting in a few cancerous cells migrating to the lungs and subsequently prompt the recruitment of CD3+ T cells to the lungs. However, these findings should be interpreted with caution, and it is recommended that future studies should allow the experimental animals to live for at least 4 months in order to adequately assess the effect of 2‐ME on pulmonary metastasis when treatment is initiated before the development of primary mammary tumor.

Among the 13 cytokines investigated, IL‐6 plasma levels were significantly lower in the 2‐ME group, while IL‐27 exhibited notably reduced levels in the treated group. Both are pleiotropic cytokines,^52^, ^53^ with elevated levels being associated with BC tumor progression, therapeutic resistance, and poor prognosis.^54^, ^55^, ^56^, ^57^, ^58^ Low levels of IL‐10, IL‐12p70, IL‐23, and IFN‐β were observed. These cytokines are all pro‐inflammatory cytokines with the exception of IL‐10 which is anti‐inflammatory.^59^ These pro‐inflammatory cytokines have anti‐tumor effects such as inhibiting BC proliferation.^60^, ^61^, ^62^ Anti‐inflammatory IL‐10 is associated with poor prognosis, and in animal models, inhibiting IL‐10 signaling hinders tumor growth.^63^ Additionally, IL‐6 functions as an anti‐inflammatory cytokine by promoting potent IL‐10 cytokine production by T cells.^64^ Moreover, Yasukawa et al showed that activated monocytes and macrophages produce IL‐10 in response to IL‐6.^65^ Both IL‐6 and IL‐10 were low in the 2‐ME group, and this could possibly be accounted for by the low CD3+ and CD163+ cell numbers observed, resulting in the lower levels of these cytokines. Although, one of the outcomes is beneficial and the other pathogenic, this shows that 2‐ME influences multiple immune cells that exhibit contradictory effect on BC progression at the molecular level. The 2‐ME treated group had higher levels of pro‐inflammatory IL‐1β compared to controls. IL‐1β is a pro‐inflammatory cytokine that promotes BC growth and is associated with poor prognosis.^66^, ^67^ Taken together, the low levels of IL‐12p70, IL‐23, and IFN‐β combined with high level of IL‐1β are likely to contribute to the tumor mass increase observed. However, the differences in these cytokines considering both the 2‐ME and control groups is not noticeable let alone significant. Therefore, when considering the notably low (IL‐6 and IL‐27) and high (IL‐10) level of cytokines, as well as lower number of CD163+ cells, 2‐ME may also have rendered an anti‐tumor effects such as the greater tumor necrosis.

The findings of this study supported the findings of our initial investigations where we found that 2‐ME treated mice also had higher tumor mass in early‐stage BC. However, this observation was not made when 2‐ME was administered to late‐stage BC animals as supported by the observation of fewer CD3+ T cells in the TME. In early‐stage BC, CD3+ T cells were similar in both control and 2‐ME treated groups. Late‐stage BC had lower tumor mass and higher CD3+ T cell numbers. These results suggest that the number of CD3+ T cells in the TME may be important. There was also a significant increase in tumor necrosis observed in late‐stage BC. Numerous studies have reported that 2‐ME is anti‐tumorigenic, decreasing or slowing tumor growth,^15^, ^18^, ^68^, ^69^ although in these studies, 2‐ME treatment was initiated after palpable tumors were present. Furthermore, these studies highlighted the anti‐tumorigenic properties of 2‐ME, demonstrating its efficacy in inhibiting BC tumor growth in non‐transgenic mice transplanted with BC cells.^18^, ^69^ Additionally, the dosage and duration of 2‐ME varied, ranging from 25 to 150 mg/kg/day and from 16 to 29 days.^15^, ^18^, ^68^, ^69^ Our results suggest that 100 mg/kg of 2‐ME treatment administered eight times may be optimal for promoting necrosis.

Based on the findings of this study, it is still unclear whether 2‐ME can be used as preventative treatment in individuals who are predisposed to BC. Additionally, the mechanism of action of 2‐ME, especially in the different stages of BC, is not fully elucidated, and more studies are required to understand the pleiotropic effect of 2‐ME in BC stages over a longer period. However, our findings do suggest that 2‐ME contributes to earlier development of mammary carcinoma, but the effect of tumor progression in this transgenic mouse model needs further investigation. If more mice were included in the study for a longer time to allow for pulmonary metastasis, a more conclusive result might have been observed. The significant increase in tumor mass could be the result of significantly fewer CD3+ T cells. However, there were fewer CD163+ cells and greater tumor necrosis in 2‐ME treated mice. Moreover, there were no other noticeable changes in the lungs except for fewer CD3+ T cells in the 2‐ME group, that could be a result of 2‐ME inhibiting T cell proliferation. Furthermore, since the time was shorter than when pulmonary metastasis is expected in this model, this result on lung metastasis should be interpreted with caution. Considering the above, the best anti‐tumor outcome was observed in mammary tumors of late‐stage BC, but not with pulmonary metastasis and longevity. Moreover, 2‐ME administered with hormone and chemotherapeutic drugs such as paclitaxel, tamoxifen and doxorubicin, has led to enhanced anti‐tumor effect.^70^, ^71^, ^72^ It is therefore possible from this study that this combination may render different effects in various stages of BC and should be considered in future experimental design.

## 5 CONCLUSION

In this study, we found that 2‐ME treatment may promote early palpable mammary tumor development and progression. The significant tumor mass increase occurred in response to protumor cellular events, such as fewer CD3+ cells and signaling changes, such as higher IL‐1β levels and lower levels of IL‐12p70, IL‐23 and IFN‐β, in the TME. Despite this, there was evidence of anti‐tumor activity such as the low number of CD163+ cells, greater necrosis, high levels of IL‐10 and low levels of IL‐6 and IL‐27. Therefore, 2‐ME promoted initiation of early tumor development, while also providing anti‐tumor activity at the molecular level.

## AUTHOR CONTRIBUTIONS

**Kimberly T. Peta:** Conceptualization; investigation; formal analysis; writing – original draft; data curation; visualization; methodology. **Chrisna Durandt:** Conceptualization; formal analysis; methodology; writing – review and editing; visualization; supervision. **Marlene B. van Heerden:** Methodology. **Anna M. Joubert:** Writing – review and editing. **Michael S. Pepper:** Conceptualization; resources; writing – review and editing; supervision. **Melvin A. Ambele:** Conceptualization; formal analysis; resources; writing – review and editing; visualization.

## FUNDING INFORMATION

Melvin A. Ambele is supported by the South African Medical Research Council Self‐Initiated Research Grant (grant no. A1A982), and by the National Research Foundation Competitive Support for Unrated Researchers (grant no. 114044); Michael S. Pepper is supported by grants from the South African Medical Research Council University Flagship Project (SAMRC‐RFA‐UFSP‐01‐2013/STEM CELLS), the SAMRC Extramural Unit for Stem Cell Research and Therapy, and the Institute for Cellular and Molecular Medicine of the University of Pretoria.

## CONFLICT OF INTEREST STATEMENT

The authors have stated explicitly that there are no conflicts of interest in connection with this article.

## ETHICS STATEMENT

The animal study received ethical approval from the Faculty of Health Sciences Research Ethics Committee (REC166‐19) and the Animal Ethics Committee (534/2019) at the University of Pretoria.

## Data Availability

The data that support the findings of this study are available from the corresponding author upon reasonable request.
